# Liquid on Paper: Rapid Prototyping of Soft Functional Components for Paper Electronics

**DOI:** 10.1038/srep11488

**Published:** 2015-07-01

**Authors:** Yu Long Han, Hao Liu, Cheng Ouyang, Tian Jian Lu, Feng Xu

**Affiliations:** 1 The Key Laboratory of Biomedical Information Engineering of the Ministry of Education, School of Life Science and Technology, Xi’an Jiaotong University, Xi’an 710049, China; 2 Bioinspired Engineering and Biomechanics Center (BEBC), Xi’an Jiaotong University, Xi’an 710049, China

## Abstract

This paper describes a novel approach to fabricate paper-based electric circuits consisting of a paper matrix embedded with three-dimensional (3D) microchannels and liquid metal. Leveraging the high electric conductivity and good flowability of liquid metal, and metallophobic property of paper, it is possible to keep electric and mechanical functionality of the electric circuit even after a thousand cycles of deformation. Embedding liquid metal into paper matrix is a promising method to rapidly fabricate low-cost, disposable, and soft electric circuits for electronics. As a demonstration, we designed a programmable displacement transducer and applied it as variable resistors and pressure sensors. The unique metallophobic property, combined with softness, low cost and light weight, makes paper an attractive alternative to other materials in which liquid metal are currently embedded.

Soft electronics has drawn considerable attention as a substitute for conventional hard devices in personal healthcare and human daily life due to its capability to match the elasticity of human tissue and preserve natural ranges of motion^1^,^2^. Amongst all substrates for soft electronics, paper has recently attracted increasing interests (known as paper electronics) due to its light-weight, soft, environmental friendly and disposable properties^3^. Besides, paper-based devices have shown great success as novel platforms for simple, portable and inexpensive devices^3^,^4^, with widespread applications in diverse areas ranging from mechanical^5^, electrical^6^ to biochemical fields^7^. Besides, the price of paper (~0.1 cent dm^−2^) is substantially lower than that of other substrates currently used for soft electronics such as polydimethylsiloxane (PDMS) and silicon^8^,^9^,^10^,^11^,^12^,^13^,^14^. Thus, there exist great economic and technological motivations for the development of paper electronics, which holds great potential for applications in digital microfluidic chips^15^, energy storage devices^16^ and flexible displays^17^.

Commonly, paper electronics are fabricated by patterning conductive materials on the surface of paper^18^,^19^,^20^. For example, conductive inks (*e.g.*, silver nanoparticles ink) have been ink-printed onto paper to fabricate various functional components^5^. This strategy offers high spatial resolution in circuit fabrication and great integrity for paper-based electronics. However, it is associated with the limitations of high cost and complex fabricating process. More importantly, paper electronics fabricated via these methods involve the issue of unstable electric behaviors (*e.g.*, conductivity and functionality), as induced by the intrinsic defects of paper including inevitable roughness and porousness^3^. Therefore, there is still an unmet need for a simple, rapid prototyping approach to fabricate paper electronics with high electric performance.

Recently, liquid-phase conductive materials have been embedded into insulative matrix for construction of soft electronics, which holds great potential to address the challenge of unstable electric function in paper electronics. Liquid metals (*e.g.*, gallium-indium alloys) offer an emerging class of highly conductive materials for embedding^21^. Compared with conventional materials in electronics such as solid metals or metal oxides, liquid metals can deform easily to match the deformation of a substrate. Due to this unique advantage, significant efforts have been devoted to fabricating structures embedded with liquid metals for soft electronics. For instance, a masked patterning and deposition method has been developed to manufacture liquid-embedded soft conductors with high spatial resolution, but this type of method often requires a mold fabricated by photolithography^22^,^23^. Recently, laser-patterning, direct writing and 3D printing approaches have also been brought up to avoid the need for replica molding and photolithography^24^,^25^,^26^. However, most of these methods involve silicon elastomers (*e.g.*, PDMS) that are not environmental friendly since they may sediment in freshwater areas^27^.

Using green materials as insulative matrix (*e.g.*, paper matrix) is promising to solve the above-mentioned problems, which has not been explored before. There are two main challenges that require to be addressed before embedding liquid metal into paper matrix. It is easy to understand that paper has porous structure, which leads to the poor barrier properties for liquid. Hence, it is critical to study the interaction of paper with liquid metal, and prevent the leakage of liquid metal from porous paper. The other is how to pattern liquid metal into paper matrix rapidly to produce 3D soft circuits with high electric performance.

In our previous work, we have developed a facile method to fabricate a hybrid composite with 3D microfluidic channels embedded with liquid metal for soft electronics^28^. Here, we hypothesize that the thin oxide skin naturally formed on the surface of liquid metal would help to prevent from wetting the paper due to the resulted high surface tension, thus avoiding leakage when embedded in paper. To test this hypothesis, we firstly investigated the wetting process of liquid metal with paper. Then we developed a platform for prototyping soft conductors composed of liquid-metal and 3D microfluidics. Further, to demonstrate the versatility of our method, we designed a programmable displacement transducer, and its applications as variable resistors and pressure sensors were also demonstrated.

## Results

### Interaction of metallophobic paper and liquid metal

**Figure 1:**
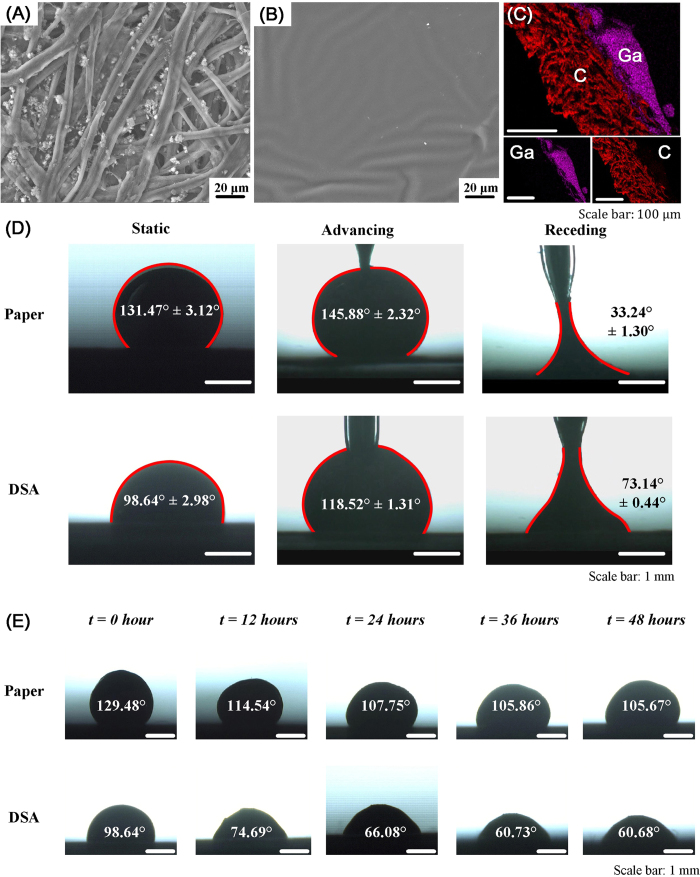
Wetting properties of liquid metal on paper. Scanning electron microscope (SEM) images illustrate the surface topological structures of cellulose paper (A) and double sided adhesive (DSA) (B). (**C**) Energy dispersive spectrometry (EDS) elemental mapping of the cross-section of a cellulose paper was used to observe the penetration of liquid metal into porous paper. C and Ga represent the element of carbon and gallium and indicate paper and liquid metal respectively. (**D**) Characterization of the static, advancing and receding contact angles of liquid metal on paper and DSA via optical images captured from corresponding videos (Supporting Information, Videos M1-M4) of sessile drops. Values demonstrated represent average measured contact angles with 95% confidence intervals. Red outline of each droplet image marks the profile of drop boundary. (E) Time-sequenced optical images show the wetting process on paper and DSA as a function of time.

Topological structure of substrate plays a vital role in the wetting behavior of liquid metal^29^, thus the topological structure of cellulose paper (as major substrate) and double sided adhesive (DSA, as bonding material of multilayered devices) were imaged using scanning electron microscope (SEM) (Fig. 1A,B). As can be seen from the SEM photos, paper presents a porous and rough surface while DSA has a smoother one. To test the penetration of liquid metal into porous paper, a liquid metal droplet was pipetted on a paper and the cross-section of paper was observed one week later using energy dispersive spectrometry (EDS) element mapping under SEM (Fig. 1C). We observed a clear boundary between the carbon and gallium elements, indicating that there is no significant penetration of liquid metal into paper. We believe that this is attributed to the oxidation layer formed on the surface of the liquid metal droplet when exposed to air. This oxide layer can stabilize the liquid metal in nonequilibrium shapes^26^,^30^, which prevents liquid metal from penetrating into the porous texture of paper. In addition, the elements of carbon and gallium were used to represent paper and liquid metal respectively since the EDS spectrum shows that they are both the major components of corresponding materials (Fig. S1).

Contact angles are commonly used to study the interaction of liquid and solid substrate. To understand the wetting process of liquid metal on paper and DSA, we measured the dynamic and static contact angles (Fig. 1D). The difference in advancing contact angles between paper (145.88 ± 2.32^°^) and DSA (118.52 ± 1.31°) indicates different spreading dynamics when liquid metal initially comes into contact with these two substrates. With respect to static contact angle, both static contact angles are larger than 90^o^, indicating low wettability (metallophobic) between both substrates and liquid metal. But liquid metal exhibits a higher level of wetting with DSA (98.64 ± 2.98^o^) compared to paper (131.47 ± 3.12^o^). With respect to receding contact angle, the liquid metal presented a larger receding contact angle on DSA (73.14 ± 0.44° ) than that on paper (33.24 ± 1.30^o^), suggesting that paper has stronger adhesion with liquid metal than DSA. Further, we also checked the changes of static contact angles of liquid metal on paper and DSA as a function of time, Fig. 1E. We observed that the wetting of liquid metal on both substrates happened on the order of hours. At the time point of 36 h, the contact angles of liquid metal on paper and DSA were stable at 105.86^o^ and 60.73^o^ respectively. These results agree well with the wetting process of liquid metal on textured surface^29^. The metallophobicity of paper and DSA benefits from the oxide layer around the liquid metal droplet, which hampers liquid metal penetration into the texture and subsequent wetting on paper, contributing to the integrality of paper electronics by preventing from leakage.

### Fabrication of paper based conductors using metallophobic paper and DSA

**Figure 2:**
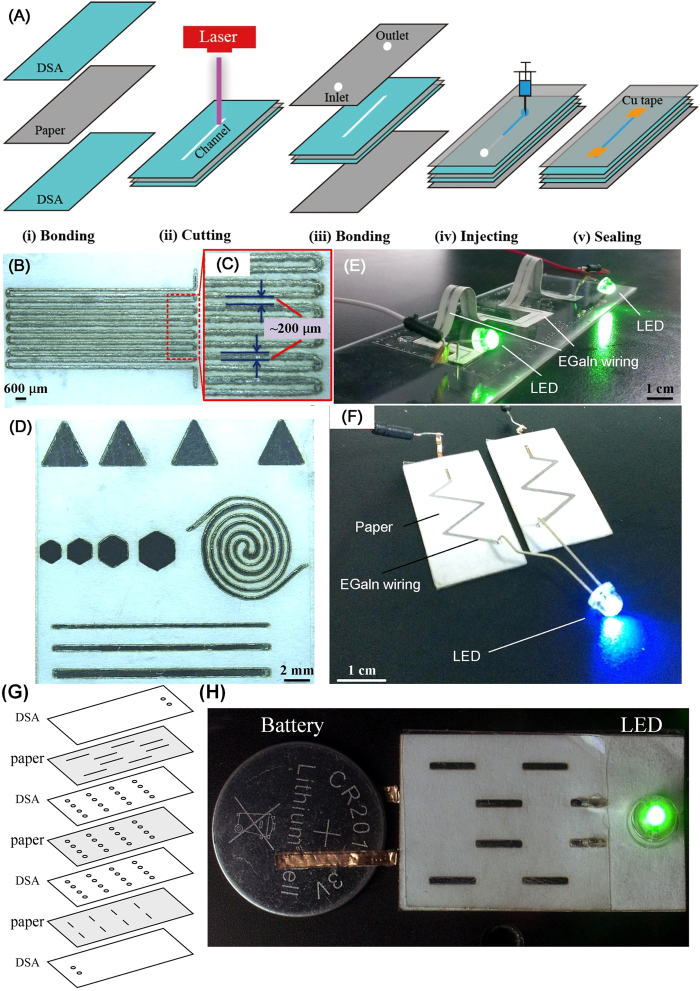
Rapid prototyping of paper-based conductors. (**A**) Fabrication steps for a paper-based conductor composed of a multilayered paper matrix and liquid metal. (**B**) Test sample of a three-layered paper-based conductor patterned with a laser spot (70 μm in diameter). Enlarged photo (**C**) shows that the patterned paper-based conductor has a minimal line width of ~200 μm and line-line distance of ~200 μm. (**D**) Patterned paper-based conductors with various two-dimensional structures. (**E**) Test sample of a “bridge” conductor made by paper origami. (**F**) LED circuit containing a paper-based conductor. (**G**-**H**) An interconnecting vertically multilayered 3D circuit containing a button battery, a LED and three layers of patterned paper.

Here, we introduced a layer-by-layer method to fabricate paper-based microfluidic electronics. A multilayered structure was first achieved by bonding DSA tapes on both top and bottom sides of a piece of printing paper (Fig. 2A(i)). Then, a customer-designed channel with specific geometry was sculptured on this 3-layer substrate utilizing CorelDraw X3 as the digital design software and laser etcher as the fast, accurate graver (Fig. 2A(ii)). Alternatively, the etching process can also be achieved manually using a knife, as demonstrated in our previous study^28^. Afterwards, protective layers of the DSA tapes were carefully removed using a tweezer, revealing adhesive surfaces that were subsequently attached to printing papers to form new top and bottom layers. Specifically, the brand new top surface possessed two etched holes as inlet and outlet (Fig. 2A(iii)) for liquid metal injection employing a syringe associated with a pump (Fig. 2A(iv)). To fulfill better visualization and observation, the top layer could also be replaced by a transparent DSA tape. The inlet and exit holes were then sealed with copper conducting tapes as electrodes connecting to the outer circuit when the injection process came to an end (Fig. 2A(v)). Regarding to the resulted microchannel, the bottom and top walls are made of paper, and the side walls are composed of both paper and DSA. Since the liquid metal will contact and interact with side wall of microchannel, the metallophobicity of DSA helps to confine the liquid metal and prevent from leakage.

To demonstrate this method, we used a laser system to fabricate a paper-based conductor with an array of liquid metal embedded. The result clearly indicates that the minimal line width is ~200 μm with line-line distance at ~200 μm (Fig. 2B,C). This spatial resolution is limited by the laser spot size (70 μm in diameter), thus smaller feature sizes could be potentially achieved by optimizing the optical path. To test the versatility of the method, we fabricated various 2D arrays using the digital laser system including lines, triangles, hexagons and a spiral (Fig. 2D). The shape and width of a pattern can be designed accurately in the software. In addition to 2D patterns, a 3D structure (i.e., bridge conductor) can be also fabricated by bending planar conductor (Fig. 2E), which paves the way for fabricating soft electric circuits into 3D structures. Last but not the least, this fabrication process is rapid, and could be accomplished within 30 min on the lab bench. To test the conductivity of our paper-based conductors, we constructed an electric circuit that composes of a power, a paper-based conductor and a LED. The result showed that the fabricated circuit has fine conductivity to light up the LED (Fig. 2F). Further, to demonstrate the ability of our method to fabricate 3D circuits, a vertically interconnecting multilayered 3D circuit is fabricated, following the idea of a traditional multilayer stacked 3D microfluidic as defined by George M. Whitesides^31^,^32^,^33^,^34^. The 3D circuit is composed of a button battery, a LED and a paper-based device with channels (Fig. 2G and H). The new device is made by aligning three layers of paper that contains planar microfluidic channel (gray layers in Fig. 2G). The top-layer microfluidic channel is vertically interconnected with the bottom-layer microfluidic channel through the holes in middle layer. These triple-layer paper device is assembled using patterned DSAs (white layers in Fig. 2G).

### Electricalmechanical stability of fabricated paper electronics

**Figure 3:**
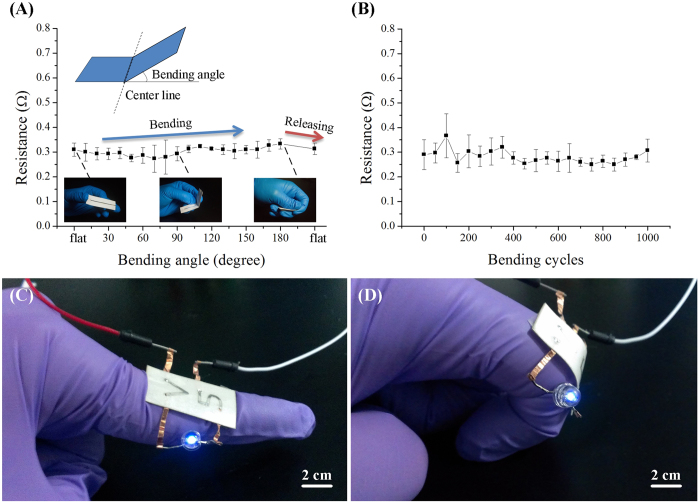
Electric stability of paper-based conductors. (**A**) Resistance variations of paper-based conductors as a function of bending angles. Top inset illustrates that the bending angle is the angle between original state and bended state. Bottom inset shows the representative bending angles at 0, 90 and 180^o^, respectively. (**B**) Resistance of paper-based conductors as a function of bending cycles at a bending angle of 90^o^. Three samples with a ~40 mm long, ~2 mm wide and ~200 μm high channel embedded with liquid metal were used in all experiments. The error bars stand for standard deviation. (**C**,**D**) The circuit is elastically compatible with finger movement from flat (**C**) to bending condition (**D**).

To further study the electric stability of our paper-based conductors against mechanical deformation, we measured the resistance of a fabricated conductor as a function of the extent of deformation, namely bending angles (Fig. 3A). We observed that the paper-based conductors remained conductive in a wide range of bending angles (0 to 180 degree) with the total resistance change of ~0.03 Ω (10%). After the release, the total resistance increased by ~0.01 Ω (3%), which may attribute to the irreversible deformation of microchannels. Surprisingly, even after 1000 bending-releasing cycles at a bending angle of 90^o^, we did not observe any significant irreversible increase in total resistance of the paper-based conductors (Fig. 3B). These results imply that the fabricated paper-based conductors possess distinguished electric stability and hold great potential for high-performance flexible conductors. In addition, the fabricated circuit is potentially elastically compatible with human movement (Fig. 3C,D).

### Design of flow-assisted programmable displacement transducer

**Figure 4:**
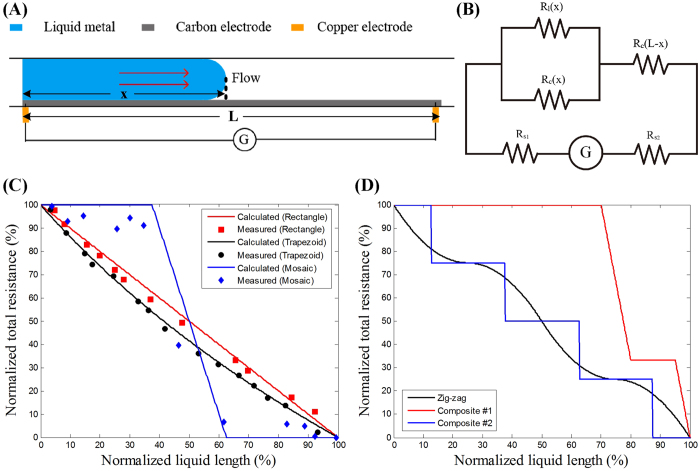
Flow-assisted programmable displacement transducers. (**A**) Schematic illustrates the working principle of a microfluidic transducer. When liquid metal flows through the channel, the total resistance changes dynamically as a function of liquid displacement. The function depends on the geometry of electrode, such as length, width and height. (**B**) Circuit diagram of the microfluidic transducer. (**C**) Measured and calculated displacement-resistance curves of microfluidic transducers with different electrode designs, *i.e.*, rectangular, trapezoidal and mosaic (*i.e.*, two-layered) electrodes. The resistance and displacement values were normalized to the maximum resistance and displacement. The channel lengths are ~40 mm and the resistance values are about 8, 6 and 3 MΩ, respectively. (**D**) Complex working curves achieved by multilayered electrodes. The details of electrode design can be found in Fig. S2.

To illustrate the robustness of our platform to fabricate functional paper electronics, we firstly came up with the concept of a flow-assisted microfluidic transducer that is capable of converting the information of liquid displacement to resistive signals. The microfluidic displacement transducer is composed of a microchannel decorated with an electrode on the bottom surface (Fig. 4A). When liquid metal flows through the channel, the total resistance changes as a function of liquid displacement. The function depends on and could be programmed by the geometry of carbon electrode (*e.g.*, length, width and thickness). Fig. 4B depicts the circuit diagram of the microfluidic transducer when the liquid metal flows to the place with a displacement of **x**. The predicted resistance is calculated as


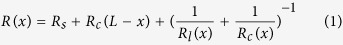


where **x** is the displacement of liquid metal in microchannel while ***L*** is the total length of the carbon electrode. The resistance value is composed of three parts. The first part is the contact resistance between copper and carbon electrodes, *i.e.*, ***R***_***s***_; The second part is the resistance of the carbon electrode that is not covered by liquid metal, *i.e.*, ***R***_***c***_***(L-x)***; The third part is the resistance of liquid-covered carbon electrode, which can be predicted according to Ohm’s Law, *i.e.*, 
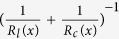
. Since *R*_*c * ≫ _*R*_*l*_, Eq.(1) can be reduced to 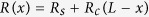. By rewriting ***R***_***c***_***(L-x)*** in terms of its volume resistivity (***ρ***) and geometry factors (*i.e.*, the width (***D(x)***) and height (***H***)), Eq.(1) becomes


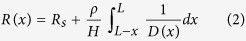


Eq. (2) lays the theoretical basis for encoding the working curve of microfluidic transducer. ***R(x)*** could be adjusted through the geometry ***D(x)***, which is determined by experimental design. For example, if the width of the electrode is constant along with the channel, the resistance ***(R)***will have a linear relationship with the displacement ***(x)***; if the width of the electrode increases linearly, the rate at which the resistance ***(R)*** changes increase linearly with length ***(x)***.

To test the theory, we firstly fabricated a rectangular and a trapezoidal carbon electrode (Fig. S2A,B), and measured the length-resistance curves, which agreed well with the calculated results (Fig. 4C). Besides the changes in electrode geometry, we also designed mosaic electrodes (*i.e.*, multilayered electrodes) with different materials (*e.g.*, copper and carbon), resulting in a new function ***ρ(x)*** that describes the changes of volume resistivity ***(ρ)*** along with the displacement ***(x)***. For a two-layered electrode made from copper and carbon (Fig. S2C),


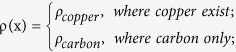


The measured and predicted values also show a strong agreement for this mosaic electrode (Fig. 4C).

In summary, all our experimental and theoretical results support that the displacement-resistance function of our microfluidic transducer could be programmed by designing the geometry of the carbon electrode. Theoretical analysis provides a tool for function prediction therefore guides device fabrication, especially when the complex resistance-displacement curves are demanded (Fig. 4D). However, the current experimental designs using carbon or copper electrodes failed to reversibly function since their strong interaction with liquid metal. This problem could be potentially conquered by the discovery of metallophobic electrodes.

### Applications of the flow-assisted programmable displacement transducer

#### Variable resistors

**Figure 5:**
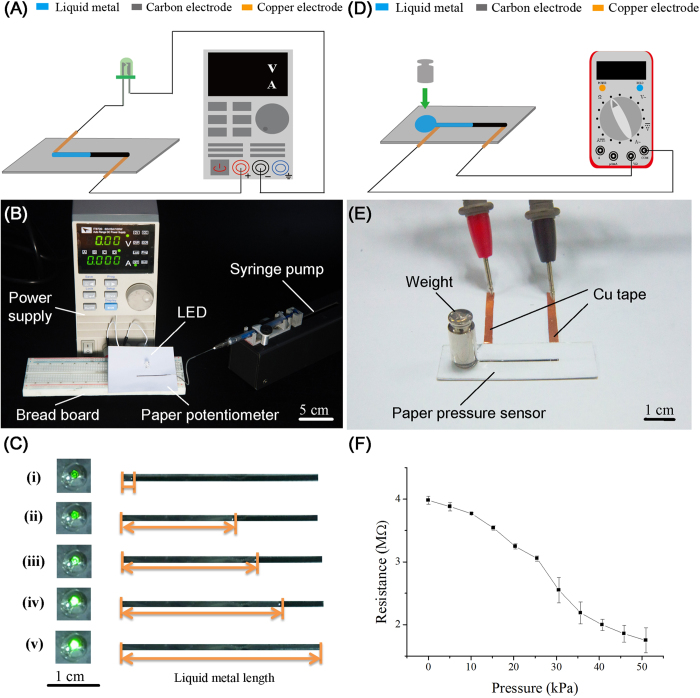
Applications of the microfluidic displacement transducer as a variable resistor and a pressure sensor. (**A**) Schematic shows the working principle of a flow-assisted microfluidic variable resistor. (**B**) Experimental setup for controlling LED intensity using a paper-based flow-assisted variable resistor. The channel used here is 40 mm in length, 2 mm in width and 260 μm in height. (**C**) Results show that the light intensity of LED correlates closely to the flow of liquid metal inside microchannel. (**D**) Schematic shows the working principle of a pressure sensor. (**E**) Image of the experimental setup used for pressure detection. The pressure sensor is composed of a storage tank (diameter of 9 mm and height of 260 μm) and a connected channel (width of 2 mm, length of 25 mm and height of 260 μm). (**F**) Pressure-resistance curve of the paper-based pressure sensor. Each point represents the average value and the error bars stand for standard deviation (n = 3).

As a demonstration of the ability to fabricate functional components for paper electronics, we prototyped a soft variable resistor on paper. Variable resistors are common electronic units that employ the relationship between displacement and resistance. Thus, based on the transducing mechanism mentioned above, we reasonably proposed the concept of programmable microfluidic variable resistor in which total resistance of the device varies as a result of the flow of liquid metal in the microchannel. To verify this, we established a simple circuit containing a variable resistor (Fig. 5A,B). The LED brightness can be controlled by the flow of liquid metal in the microchannel (Fig. 5C), which further indicated that resistance shifted as a function of displacement of the liquid metal.

#### Pressure sensor

Flexible sensors are one of significant applications of liquid metal^35^,^36^. Prof. Wood’s group laid the cornerstone of wearable devices for multifunctional sensors^37^. Pressure sensor is among the most extensively used sensors in technology and industry areas. Here, we fabricated a simple, low-cost and disposable pressure sensor (Fig. 5D,E). In our design, a storage tank was etched on printing paper connected to the microfluidic channel. Accordingly, when pressure was applied on the storage tank full of liquid metal, the conductive liquid was forced to flow into the adjacent channel, resulting in the changes of liquid displacement and total resistance. Experimental results indicated that this design was feasible and with good pressure sensitivity (approximately 5 kPa) (Fig. 5F). This portable and disposable paper-based device have wide applications for force/pressure measurement.

## Discussion

We demonstrate that the liquid metal could be patterned on paper with a laser etcher to produce soft electrodes and sensors. The desirable fabrication approach should be rapid, cost-effective, and easy to access with high spatial accuracy and capability of prototyping 2D and 3D conductors. The method developed here does not need any complex fabrication system, and all the steps can be processed on benchtop within 30 min. The spatial resolution of this method could achieve as high as 200 μm, which is comparable to other methods including direct writing^25^ and laser printing^24^. Meanwhile, this strategy offers excellent eco-friendly superiority via employing green paper as soft substrate material, instead of commonly used PDMS. Besides, 3D structures and 3D circuits can be fabricated by paper origami and stacking multilayer paper respectively.

Soft electronics has received a great amount of research attention since it offers possibilities for applications that cannot be achieved with rigid circuits^8^,^9^,^10^,^11^,^12^,^13^,^14^. Recently, paper electronics primarily offers the dominances of disposability, portability, and significantly lower costs over other soft electronics counterparts. Commonly, it is necessary to employ a selective technology to convert paper from hydrophilic to hydrophobic due to the poor barrier properties to liquid. For example, Whitesides *et al.* rendered paper hydrophobic using chemical modification, and deposited conductive inks on the surface of modified paper for paper electronics^38^. In contrast, we explore the inherent property of paper (*i.e.*, metallophobic), and directly embed liquid metal into 3D microfluidics to form soft circuits. This approach not only avoids the modification of paper, but also addresses the challenges of unstable electrical function induced by surface roughness of paper.

The paper-based microfluidic transducer offers a novel mechanism to convert displacement information of liquid into electric signals when liquid flows through a microchannel. The working curve of the paper-based transducer (*i.e.*, resistance-displacement curve) could be programmed into desired types by shaping different geometries of electrodes. Theoretically, any incensements in the displacement of liquid (**△x**) would lead to a variation in resistance (**△R**), and the **△R** depends on the geometry of electrodes in the range of **△x**. Thus the sensitivity could also be adjusted by geometry design. For instance, with the same **△x**, an electrode with narrower width will result in a larger **△R**, namely, higher sensitivity. Existing literatures have shown that the liquid metal is able to flow through a 20 μm wide microchannel^21^, therefore the sensitivity could be adjusted in a large range, adaptive to various applications. However, for certain applications such as a pressure sensor, the sensor sensitivity is also significantly affected by how liquid flows inside the microchannel driven by an applied pressure. This transformation of pressure signals into displacement of liquid involves a complex hydrodynamic process, which is affected by numerous factors including the properties of liquid and the geometry of microchannel^21^. Thus, the sensitivity of such type of displacement transducers should be further investigated experimentally in certain cases.

## Conclusions

In summary, we demonstrated the fabrication of simple, low-cost, flexible, and disposable paper-based conductors for paper electronics. In contrast to existing paper electronics, this was accomplished by an innovative strategy, where the liquid metal was injected into paper-based device with predesigned 3D microchannels. Various 2D and 3D structures, with 200 μm level widths and inter-line spacing, were made using unmodified paper and double sided adhesive, which can be achieved in 30 min on lab bench. We investigated the wetting process of liquid metal on paper and concluded that the paper is metallophobic with a static contact angle at 131.47 ± 3.12^o^. Then we presented that the fabricated soft paper-based conductors could maintain their electronically mechanical stability both at different bending angles from 0 to 180^o^, and after 1000 cycles of bending deformation with a bending angle of 90^o^. Finally, as an example, we demonstrated the concept of microfluidic displacement transducer. Theoretical and experimental results suggested that the working curve and sensitivity of the transducer could be programmed through the design of electrodes. This small, low-cost, disposable paper-based device holds great potential to be used in many applications such as flexible sensors and paper electronics.

## Methods

### Characterization of wetting process

The contact angle measurements on 2 different substrates were achieved employing the sessile drop method. A static sessile drop (3 μL) were generated by directly pipetting EGaIn (495425, Sigma Aldrich) on a paper and a DSA. The static sessile drops were observed and imaged immediately using a digital microscope (VHX-600, Keyence) with a 50X objective, and the static contact angles were further quantified using Image Pro Plus (IPP) from images of the static sessile drop. To characterize dynamic contact angles, we used a syringe pump (TJ 1A/L0107-1A, Longer Pump) to add (or withdraw) EGaIn into (or from) the 3 μL static sessile drop at a flow rate of 30 μL/min. The advancing and receding processes of the sessile drop were recorded using the digital microscope. The dynamic contact angles were quantified from the images extracted from the videos (Supporting Information, Videos M1-M4) using IPP. To characterize the changes of static contact angle as a function of time, the static contact angles of a sessile drop were measured every 12 h with the same method.

### Fabrication of paper electronics

Patterns were defined with accurate shapes and precise sizes by a professional graphic design software, CorelDraw X3. Etching operation on paper was achieved via a CO_2_ laser engraver (VLS 2.30, Universal Laser System) with 100% energy, 70% speed and 1000 PPI. The cutting process was repeated 2 times to ensure the channels were thoroughly through and with designed geometry. Then injection of EGaIn was performed utilizing the micro syringe pump at a flow rate of 10 μL/min till the microfluidic channels were full of EGaIn. 3D structures were obtained by further steps of bending and attaching onto a PMMA (polymethylmethacrylate) substrate.

### Flexibility characterization

The flexibility test was operated through bending the paper-based conductor along its geometrical center line with bending angles ranging from 0° to 180°, and resistance measurements were implemented every 10° bending angle. Subsequently, the paper conductor was straightened and corresponding resistance was obtained via a multimeter (UT805, UNI-T). The fatigue test was achieved by manually bending the paper conductor to a 90° bending angle and then straightening for each cycle, and resistance measurement was carried out every 50 cycles.

### Fabrication of displacement transducer

The flow-assisted displacement transducer was constituted of a paper-based microfluidic channel etched by the laser etcher and a thin piece of carbon double-sided conducting tape (FN731-5, Nissin) with unique geometrical properties placed on the bottom surface working as an electrode. The programmability of this device was achieved through designing electrodes with various shapes and sizes (Supporting Information, Fig. S2) in CorelDraw X3 and sculpturing them out via the laser etcher.

### Fabrication of variable resistor

The variable resistor was achieved based on the mechanism of above-mentioned flow-assisted displacement transducer. A light emitting diode (LED) and a direct current power supply (IT6720, ITECH) were wired to the EGaIn-contained channel via Cu electrodes. EGaIn was pumped into the channel through the syringe pump at a flow rate of 30 μL/min, and the brightness of LED changed along with the advancing flow of EGaIn in microchannel (Supporting Information, Video M5).

### Fabrication of pressure sensor

To fulfill this need, a circular storage tank with a diameter of 9 mm was first etched on printing paper, connected to the microfluidic channel (width of 2 mm, length of 25 mm and height of 260 μm). Accordingly, when pressure was applied on the storage tank full of liquid metal, specifically by using weights vary from 10 g to 200 g as pressure source applied through a 7 mm-diameter round PMMA spacer, the conductive liquid was forced to flow into the adjacent channel, resulting in changes of resistance. The corresponding resistance change was detected by the multimeter.

## Additional Information

**How to cite this article**: Han, YL. *et al.* Liquid on Paper: Rapid Prototyping of Soft Functional Components for Paper Electronics. *Sci. Rep.* **5**, 11488; doi: 10.1038/srep11488 (2015).

## Supplementary Material

Supporting Information

Supporting Information M1

Supporting Information M2

Supporting Information M3

Supporting Information M4

Supporting Information M5
