# Supplementary material for: Liquid on Paper: Rapid Prototyping of Soft Functional Components for Paper Electronics
Source: Sci Rep. 2015 Jul 1;5:11488. doi: 10.1038/srep11488 (PMC4486998; doi:10.1038/srep11488)
Supplement: Supporting Information [file srep11488-s1.pdf]

Supporting Information

Liquid on Paper: Rapid Prototyping of Soft Functional Components for Paper Electronics

Yu Long Han[1,2#], Hao Liu[1,2#], Cheng Ouyang[1,2], Tian Jian Lu[2*], Feng Xu[1,2*]

[1] *The Key Laboratory of Biomedical Information Engineering of the Ministry of Education, School of Life Science and Technology, Xi'an Jiaotong University, Xi'an 710049, China*

[2] *Bioinspired Engineering and Biomechanics Center (BEBC), Xi'an Jiaotong University, Xi'an 710049, China*

[#] *These authors contributed equally to this work*

[*] *Corresponding authors:* tjlu@mail.xjtu.edu.cn, fengxu@mail.xjtu.edu.cn

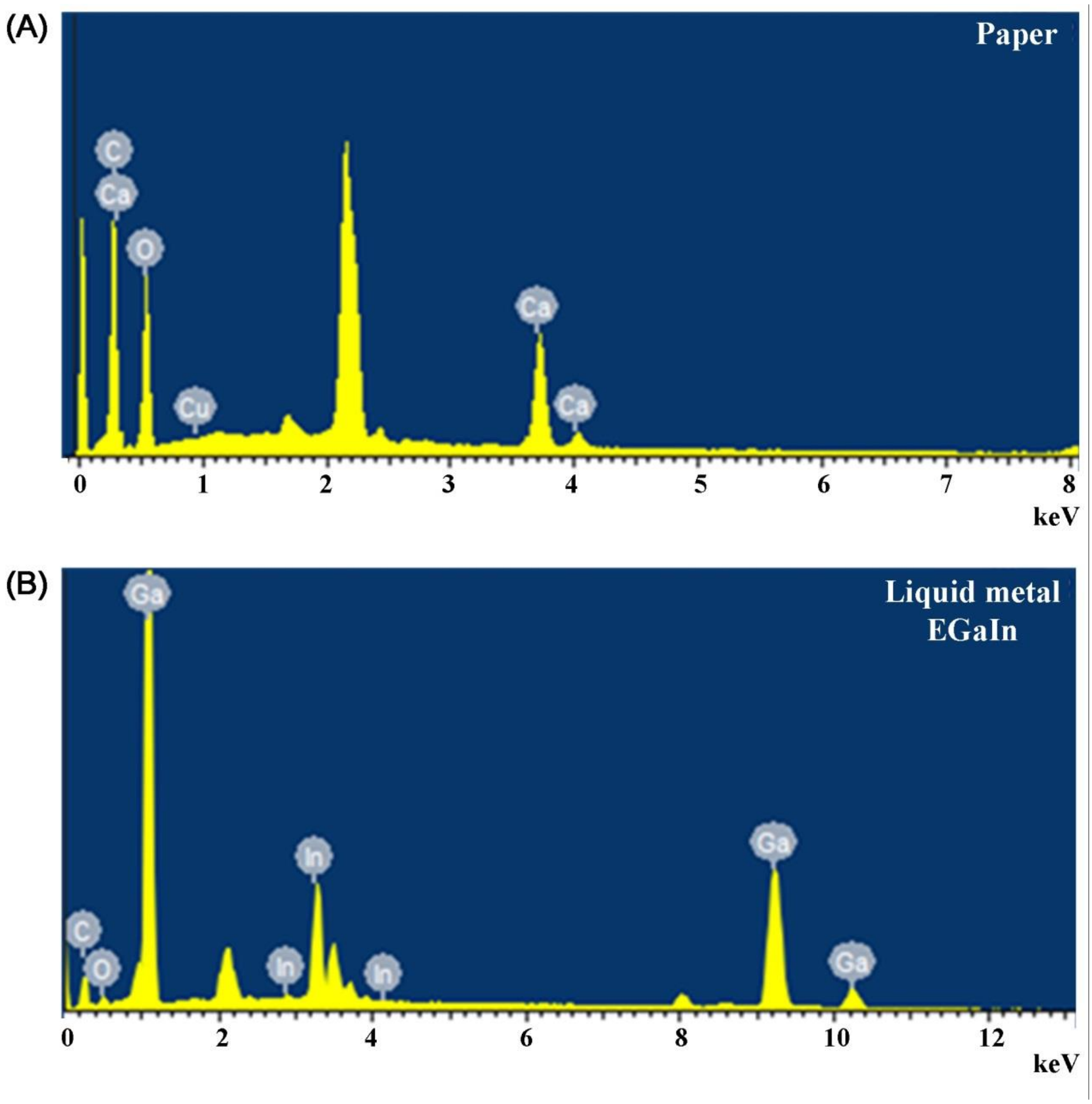

Figure S1. Energy dispersive spectrometry (EDS) analysis of paper (A) and liquid metal (B). The EDS spectrum of paper shows four main elements, including C, O and Ca. The EDS spectrum of EGaIn shows two main elements, including Ga and In.

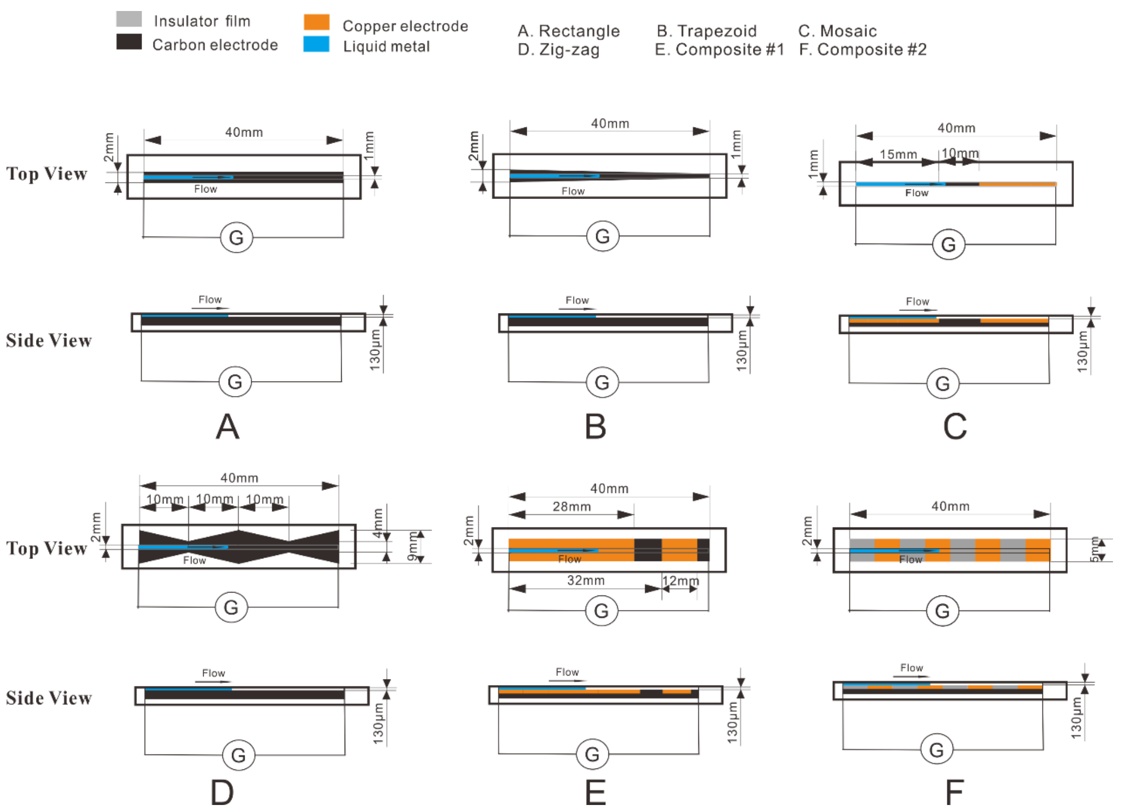

Figure S2. Schematic shows the design of electrodes in flow-assisted programmable displacement transducers. (A) A rectangular electrode. (B) A trapezoidal electrode. (C) A mosaic electrode made of copper and carbon. (D) A zig-zag electrode. (E) Composite #1: a two-layered electrode made of copper and carbon. (F) Composite #2: a two-layered electrode made of carbon, copper and insulator.

Experimental Section

Characterization of Wetting Process: The contact angle measurements on 2 different substrates were achieved employing the sessile drop method. A static sessile drop (3 μL) were generated by directly pipetting EGaIn (495425, Sigma Aldrich) on a paper and a DSA. The static sessile drops were observed and imaged immediately using a digital microscope (VHX-600, Keyence) with a 50X objective, and the static contact angles were further quantified using Image Pro Plus (IPP) from images of the static sessile drop. To characterize dynamic contact angles, we used a syringe pump (TJ 1A/L0107-1A, Longer Pump) to add (or withdraw) EGaIn into (or from) the 3 μL static sessile drop at a flow rate of 30 μL/min. The advancing and receding processes of the sessile drop were recorded using the digital microscope. The dynamic contact angles were quantified from the images extracted from the videos (Supporting Information, Videos M1-M4) using IPP. To characterize the changes of static contact angle as a function of time, the static contact angles of a sessile drop were measured every 12 h with the same method.

Fabrication of Paper Electronics: Patterns were defined with accurate shapes and precise sizes by a professional graphic design software, CorelDraw X3. Etching operation on paper was achieved via a CO_2 laser engraver (VLS 2.30, Universal Laser System) with 100% energy, 70% speed and 1000 PPI. The cutting process was repeated 2 times to ensure the channels were thoroughly through and with designed geometry. Then injection of EGaIn was performed utilizing the micro syringe pump at a flow rate of 10 μL/min till the microfluidic channels were full of EGaIn. 3D electronics were obtained by further steps of folding and attaching onto a PMMA (polymethylmethacrylate) substrate.

Flexibility Characterization: The flexibility test was operated through bending the

paper-based conductor along its geometrical center line with bending angles ranging from 0 ° to 180 °, and resistance measurements were implemented every 10 ° bending angle. Subsequently, the paper conductor was straightened and corresponding resistance was obtained via a multimeter (UT805, UNI-T). The fatigue test was achieved by manually bending the paper conductor to a 90 ° bending angle and then straightening for each cycle, and resistance measurement was carried out every 50 cycles.

Fabrication of Displacement Transducer: The flow-assisted displacement transducer was constituted of a paper-based microfluidic channel etched by the laser etcher and a thin piece of carbon double-sided conducting tape (FN731-5, Nissin) with unique geometrical properties placed on the bottom surface working as an electrode. The programmability of this device was achieved through designing electrodes with various shapes and sizes (Supporting Information, Fig. S2) in CorelDraw X3 and sculpturing them out via the laser etcher.

Fabrication of Potentiometer: The potentiometer was achieved based on the mechanism of above-mentioned flow-assisted displacement transducer. A light emitting diode (LED) and a direct current power supply (IT6720, ITECH) were wired to the EGaIn-contained channel via Cu electrodes. EGaIn was pumped into the channel through the syringe pump at a flow rate of 30 μL/min, and the brightness of LED changed along with the advancing flow of EGaIn in microchannel (Supporting Information, Video M5).

Fabrication of Pressure Sensor: To fulfill this need, a circular storage tank with a diameter of 9 mm was first etched on printing paper connected to the microfluidic channel (width of 2 mm, length of 25 mm and height of 260 μm). Accordingly, when pressure was applied on the storage tank full of liquid metal, specifically by using weights vary from 10 g to 200 g as pressure source applied through a 7 mm-diameter round PMMA spacer, the conductive liquid was forced to flow into the adjacent

channel, resulting in changes of resistance. The corresponding resistance change was detected by the multimeter.
